# Nanofluids for Direct-Absorption Solar Collectors—DASCs: A Review on Recent Progress and Future Perspectives

**DOI:** 10.3390/nano13071232

**Published:** 2023-03-30

**Authors:** Hussein Sayed Moghaieb, Vincenzo Amendola, Sameh Khalil, Supriya Chakrabarti, Paul Maguire, Davide Mariotti

**Affiliations:** 1 School of Engineering, Ulster University, 2-24 York Street, Belfast BT15 1AP, UK; 2 Dipartimento di Scienze Chimiche, Universita’ degli Studi di Padova, Via Marzolo 1, 35131 Padova, Italy

**Keywords:** nanofluids, solar thermal energy conversion, direct-absorption solar collectors

## Abstract

Owing to their superior optical and thermal properties over conventional fluids, nanofluids represent an innovative approach for use as working fluids in direct-absorption solar collectors for efficient solar-to-thermal energy conversion. The application of nanofluids in direct-absorption solar collectors demands high-performance solar thermal nanofluids that exhibit exceptional physical and chemical stability over long periods and under a variety of operating, fluid dynamics, and temperature conditions. In this review, we discuss recent developments in the field of nanofluids utilized in direct-absorption solar collectors in terms of their preparation techniques, optical behaviours, solar thermal energy conversion performance, as well as their physical and thermal stability, along with the experimental setups and calculation approaches used. We also highlight the challenges associated with the practical implementation of nanofluid-based direct-absorption solar collectors and offer suggestions and an outlook for the future.

## 1. Introduction

**Figure 1:**
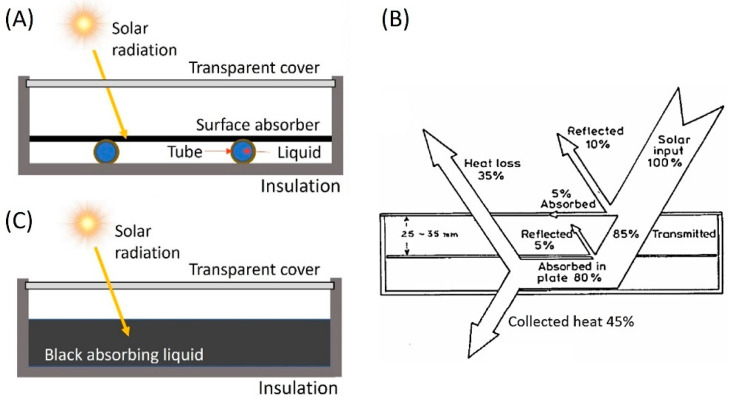
(**A**) Surface-based absorption solar collector with (**B**) the flow of both heat and solar light in a typical liquid flat plate collector. (**C**) Direct (volume-based) absorption solar collector (DASC). Reproduced with permission from [19].

Researchers from a wide range of fields are engaged in research to find solutions to meet the expected rise in renewable energy demands as a result of the growing impact of climate change and the rapid depletion of primary energy resources [1,2,3]. Since solar energy is free, clean, abundant, sustainable, renewable, nonlocalized, and generally has no net negative environmental impacts, it is being adopted at a rapid rate, increasing by 8.3% annually [3,4,5]. Governments, therefore, support technologies that can harvest solar energy efficiently and it is anticipated that by 2100, solar energy will account for nearly 70% of all global energy consumption [6,7]. Solar energy can be utilized in different forms, most notably solar-to-electrical (photovoltaic), solar-to-chemical (photosynthesis) and solar-to-thermal (photothermal) energy conversion [8,9]. For solar-to-thermal energy conversion (STC) systems, solar thermal collectors are widely used to deliver the generated thermal energy to a variety of domestic and industrial applications, such as solar refrigeration, space heating or cooling and electricity generation [6,10,11,12,13]. The traditional types of solar thermal collectors rely on solid absorbers for harvesting and converting sunlight into heat, which is then thermally transferred to working fluids, typically water, glycols, or thermal oils (Figure 1A). At the elevated surface temperature of the absorber, heat is lost at significant rates via conduction to other components of the collector, as well as convection and radiation to the environment, reducing the overall efficiency of the collector (Figure 1B). This is further complicated by issues with corrosion and chemical material degradation, which shorten the lifetime of collectors, and the absorber weight impacts installation and maintenance [14,15]. The idea of direct-absorption solar collectors (DASCs), Figure 1C, was put forth to completely remove the solid absorber and directly expose the working fluid to solar radiation for it to be absorbed and converted into heat in a volumetric STC process, overcoming the issues associated with surface-based collectors [16,17,18]. DASCs also enable the development of novel and adaptable configurational arrangements without being constrained by the incorporation of multiple components in conventional collectors, such as piping systems and surface absorbers.

Whilst solar energy is distributed over three main spectral regions (5% from the ultraviolet 300–400 nm, 43% from the visible 400–700 nm, and 52% from the near-infrared 700–2500 nm), common working fluids in solar collectors such as water, glycols, and thermal oils are good absorbers in the ultraviolet (UV) and infrared (IR) regions, both making up 57% of the total solar energy. However, they are transparent in the visible (Vis) region, meaning that ~43% of the solar energy is not efficiently captured and is instead lost as transmitted light. For instance, Otanicar et al. [20] measured the transmittance of four of the most common heat transfer liquids (water, ethylene glycol, propylene glycol, and Therminol VP-1) over a wavelength range of 200–1500 nm for a sample thickness of 10 mm. They found that water exhibited the highest absorption compared to the other fluids but could only absorb up to 13% of the incoming solar energy. This is made worse by their poor thermal conductivities [21]. In earlier attempts, black dyes were added to these conventional working fluids to improve STC performance; however, this led to the light-induced and thermal degradation of the dyes at high operational temperatures [17,22]. Suspending small solid particles with dimensions in the order of the millimetre and micrometre ranges in conventional fluids was also investigated owing to the high thermophysical and optical properties of solids compared with fluids [23]. However, DASCs suffered several operational issues such as accumulation of the suspended particles that resulted in low stability and surface erosion of pipes as well as the need for more pumping power to overcome the pressure drop brought on by the increase in friction [21].

Thanks to the rapid advances in nanotechnology, a new generation of smaller solid particles with dimensions << 1 µm (referred to as nanoparticles, NPs) was offered for their dispersion in conventional heat transfer liquids, forming nanofluids (NFs) [24]. In 1993, Masuda et al. [25] measured the thermal conductivity of three different types of metal oxide NPs suspended in water as the base fluid and they reported significant improvements in the thermal conductivity. Later, in 1995, the term nanofluid was first introduced by Choi and Eastman [26] after they worked on a copper–water NF and reported a remarkable increase in thermal conductivity over the base fluid. Since then, research on NFs has seen rapid growth in different aspects that are related to the synthesis of NPs and the preparation techniques of the corresponding NFs. Initially, NFs were investigated with respect to their thermal properties and their heat transfer characteristics. The study of their solar absorption characteristics and stability for their use in DASCs is far more recent [27,28,29]. NPs made of metals, metal oxides, and carbons (e.g., carbon black, graphene, carbon nanotubes, and carbon nanofibers) can be synthesized in different morphologies including fibres, tubes, spheres, and other shapes offering a wide variety of different types of nanoscale materials [30,31,32]. The synthesis of NPs to be used for STC systems can rely on several techniques that have found wide applicability. In this case, NP synthesis and NF preparation can be viewed as two distinct consecutive steps [33,34]. However, NPs can be also synthesized directly in the base fluid using methods such as chemical reduction in liquids [35] or physical vapour condensation [36], which require a high level of control and post-synthesis processing [37]. Generally, the overall performance of NF-based DASCs is mainly determined by the design and materials selection of the collector, and more importantly, by the characteristics of the utilized NFs.

This review is focused on NPs and corresponding NFs, their properties with relevance for DASCs, including their optical properties, resulting in STC performance and stability, with emphasis on experimental work. We have included relevant discussions on the characterization of both optical properties and stability. Important figures of merit are also discussed, which require increased attention to achieve effective and efficient STC and long-term stability under various operating conditions. The review covers the fundamentals of these parameters and various methods for measuring them before analysing the most recent progress made in research over the past few years. The review, therefore, provides a new materials science and physics perspective, that departs from relevant reviews available in the literature, and where material properties are directly linked to the NF performance. It offers an assessment of energy conversion paths, also providing the experimental methodologies to assess light–NF interactions and discuss quantitative fundamental measurements that can be used for full-scale and DASC system simulations.

## 2. Nanoparticles Used for DASC Nanofluids

### 2.1. A Classification of Nanoparticles Used for Nanofluids

A diverse range of nanomaterials has been investigated for their potential use in NFs for DASCs. It is useful to classify these with respect to their material composition and, for instance, consider metal, metal oxide, and carbon-based NPs [38,39]. Metal and carbon-based NPs generally exhibit strong absorption across the solar spectrum and specifically in the visible range; however, they often present issues in terms of stability (e.g., agglomeration and chemical degradation, and especially oxidation in high-temperature DASCs) [40,41]. Metal NPs can, in some cases, exhibit plasmonic effects, which enhance absorption in narrow spectral ranges [42]. Metal oxides are materials that offer exceptional chemical stability in a wide range of conditions and an overall reduced environmental impact; however, due to their wide energy bandgap, the optical properties can be unsuitable for solar energy absorption [43,44]. Various doping and defect engineering strategies can be used to enhance metal oxide optical properties; these can be used to lower the bandgap or to Increase the carrier density and introduce plasmonic behaviour [45,46]. Semiconducting NPs with lower bandgap values than metal oxides could, in principle, offer more favourable optical properties compared to metal oxides. However, semiconducting NPs have attracted very limited interest in DASC applications, possibly due to costs or toxicity of the raw materials (e.g., Ga, Pb, and Ge), complex and expensive synthesis methodologies (e.g., for nitrides) as well as limited stability and their propensity for chemical degradation (e.g., Si oxidation). 

The synthesis of NPs and dispersion methods in NFs can vary depending on the material and the chosen technique, which significantly impacts the resulting NF properties [47]. Methods such as chemical reduction, seed-mediated growth, sol–gel, hydrothermal, and precipitation can be used for synthesizing plasmonic metals and metal oxides [48,49]. Carbon-based nanomaterials can be synthesized through chemical vapour deposition (CVD), the Hummers method, and hydrothermal synthesis [50,51]. After synthesis, NPs can be dispersed into the base fluid using ultrasonication, magnetic stirring, or high-speed stirring and surfactants, and stabilizing agents or gum Arabic can be added to ensure stability and prevent agglomeration or sedimentation [41,52]. While NPs dispersed in the base fluids are often made from the same type of NP, i.e., single-component NF, multicomponent NFs have also been widely investigated, where the NPs in the NF are made of different materials and/or have different sizes, and/or shapes. While there are initial claims of broader absorption features, multicomponent NFs have shown to add nonnegligible complexity to the NF preparation with limited benefits [53,54].

In this section, we highlight examples of metal, metal oxide, and carbon-based NPs used in single-component as well as multicomponent NFs. A list of relevant work is also summarized in Table 1.

### 2.2. Metallic Nanoparticles

Plasmonic metals, including gold, silver, and copper, hold great promise for application in DASCs due to their inherent plasmonic absorption properties, which facilitate more efficient light absorption and scattering compared to bulk materials [8]. These metals are characterized by the presence of free electrons in their conduction bands. The collective oscillation of these free electrons in response to an electromagnetic field is known as a plasmon, which confined within an NP results in a localized surface plasmon. After absorbing light energy, plasmons can transfer energy to the lattice structure of the metal via electron–phonon coupling. resulting in the generation of heat [55]. Factors such as size, shape, and material composition of plasmonic nanostructures considerably influence their efficiency in light-to-heat conversion [56]. Additionally, the narrow bandwidth of the plasmonic effect can negatively impact the application of plasmonic NPs in DASCs, and the simultaneous excitation of transversal and longitudinal modes should be preferred in DASC NFs (e.g., using asymmetric nanostructures) [57].

**Figure 2:**
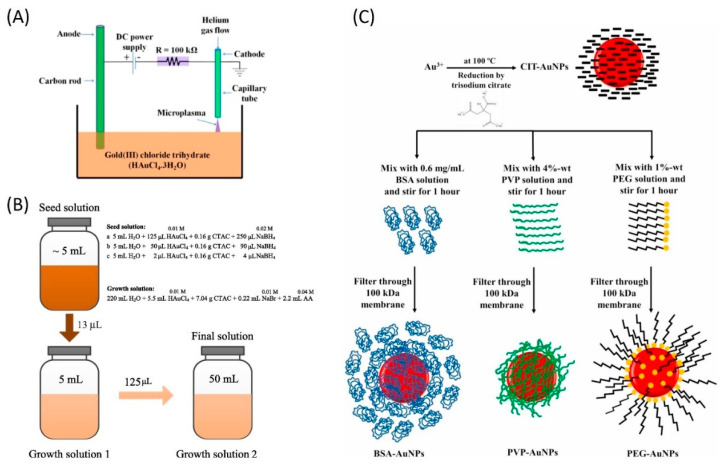
Three different methods of Au NP synthesis with and without surface modifications. (**A**) Atmospheric-pressure microplasma setup, adapted from [61]. (**B**) Seed-mediated synthesis method, adapted from [58]. (**C**) Polymer coating procedure of Au NPs prepared by chemical reduction, adapted from [60].

Numerous recent studies have explored the synthesis of metal NPs for DASCs, emphasizing their potential to boost the efficiency of solar thermal collectors. In a study that employed surface modification, Chen et al. [58] generated Au NPs with sizes of 25 nm, 33 nm, and 40 nm using a seed-mediated synthesis method, as depicted in Figure 2B. Through a series of wet chemical reactions, ultrasonication, and heating, an aqueous solution of hAuCl_4_ was reduced to Au NPs using sodium borohydride (NaBH_4_) as a reducing agent and cetrimonium chloride (CTAC) as a stabilizer. The particles from each sample were collected via centrifugation at 8000 revolutions per minute (rpm) for 10 min and were re-dispersed in water. In a recent study by Gupta et al. [59], spherical gold Au NPs with a size of ~30 nm were synthesized using the Turkevich method. Chloroauric acid was boiled and a trisodium citrate dihydrate solution was gradually added, resulting in a colour change from transparent to red, indicating Au NPs formation. After allowing the solution to cool, polyvinylpyrrolidone (PVP) was added and the mixture was stirred for 10 h to create more stable PVP-capped Au NPs. Concurrently, an Azadirachta indica leaf extract was prepared by boiling leaves in ethanol. Three heat transfer fluids were then formulated: one containing Au NPs in deionized water, one combining Au NPs with the natural extract, and the last one incorporating only the natural extract in deionized water. These NF samples were subsequently compared in a DASC for effective temperature gain and photo-thermal conversion efficiency. Sharaf et al. [60] examined the impact of four distinct surfactants on Au NPs prepared through a chemical reduction method, as shown in Figure 2C. They first synthesized citrate-capped Au NPs (CIT–Au NPs) with an average size of 13 nm by reducing hAuCl_4_ with trisodium citrate. Subsequently, they coated the CIT–Au NPs with three different polymers: bovine serum albumin (BSA), polyvinylpyrrolidone (PVP) and polyethylene glycol (PEG). After filtering the samples using a stirred cell to remove excess polymer molecules, they diluted them to achieve a concentration of 0.16 mg/mL for each NF. As surfactants can add complexity to the synthesis method and complicate heat transfer at the NP–fluid interface, McGlynn et al. [61] produced Au NPs by plasma-induced nonequilibrium electrochemistry from an aqueous gold salt precursor (hAuCl_4_.3H_2_O) without utilizing reducing or stabilizing agents, as illustrated in Figure 2A. The Au NPs were then formed into nanofluids (NFs) by drying and re-dispersing them in ethylene glycol (EG) using sonication at a concentration of 0.0055 vol%. A variety of shapes, including spherical, hexagonal, and nanorods, were observed among the Au NPs, with a mean size of 27 nm. Kimpton et al. [62] synthesized Ag NPs through a reducing–oxidizing method using citrate and PVP to achieve electrostatic and steric stabilization. Subsequently, they coated the Ag NPs with silica (SiO_2_) using tetraethyl orthosilicate (TEOS) in a basic ethanolic solution, resulting in SiO_2_@Ag NPs. The final solution was diluted with water to yield 6 mL of SiO_2_@Ag NPs.

### 2.3. Carbon-Based Nanomaterials

Carbon-based nanomaterials, such as carbon nanotubes, graphene, and graphite, exhibit strong light absorption due to their electronic structure and high aspect ratio. These materials possess a conjugated π-electron system that facilitates the free movement of electrons. Light absorption results in the excitation of electrons to higher energy states and the subsequent relaxation of these electrons release energy as heat. In addition, the strong electron–electron interactions and the unique band structure lead to the generation of nonequilibrium hot carriers. The energy of these hot carriers can be transferred to the lattice, generating heat and contributing to solar thermal conversion. The efficiency of light-to-heat conversion in carbon-based nanomaterials is influenced by factors such as material structure, size, and surface properties [24,29,63,64,65,66].

Recently, McGlynn et al. [67] produced carbon nanotubes (CNTs) using a floating-catalyst chemical vapour deposition system, which generated ribbon-like macroscopic assemblies of continuous length. The synthesis process entailed flowing ferrocene, thiophene, and methane through a furnace, with hydrogen serving as a carrier gas. Following cooling, a dense carbon aerogel formed, which was drawn into a long black “sock”. Specific gas flows and furnace temperatures were maintained to obtain ribbon-like CNT assemblies, which were then pressed between glass slides to create flat ribbon-like materials. Prior to plasma functionalization, the CNT ribbon samples were annealed in a standard air atmosphere. A plasma-induced nonequilibrium electrochemistry system was employed for the functionalization of the CNT ribbons, utilizing direct-current microplasma generated in helium between a nickel capillary tube and the electrolyte surface. The CNT ribbons underwent treatment at a constant current for 15 min and two distinct plasma–liquid treatments were investigated: one consisting of a 1:9 ethanol:water ratio and another containing 10% ethylenediamine in the same stock solution. Following plasma treatments, the ribbons were rinsed in distilled water and disentangled by sonication to form NFs. Yu et al. [66] prepared carbon-based nanofluids using a modified vortex trap method, which utilizes water mist to collect carbon NPs produced by oxygen–acetylene combustion, equipped with an external circulation device that improved carbon NP collection efficiency and production rate. In a separate study, Chen et al. [68] prepared carbon quantum dots and dispersed them in PEG-200 using a microwave heating method. They employed a 700 W home-used microwave oven to heat 5 mL of PEG-200. This heating process led to the breakdown of PEG chains, which subsequently oxidized into various fragments in the presence of oxygen, ultimately forming a surface passivation layer. The degree of carbonization was controlled by adjusting the microwave heating duration. Guo et al. [69] prepared three NFs of graphene oxide (GO), multiwalled CNTs, and Ti_3_C_2_ in 1-Butyl-3-methylimidazolium tetrafluoroborate. Commercial samples of GO and CNTs were used, while Ti_3_C_2_ was synthesized using a solvent etching method. Briefly, they dissolved 8 g of lithium fluoride in 100 mL of HCl solution (10 M) and added 5 g of Ti_3_AlC_2_ MAX to polyethylene while stirring in a water bath. The final suspension was stirred for two days and washed with water, and a thin layer of Ti_3_C_2_T_x_ was collected after multiple centrifugations and ultrasonication steps. In an extensive study on CNTs, Mesgari et al. [70] chemically functionalized single-, double-, and multiwalled CNTs and produced three different NFs with water, propylene glycol (PG), and Therminol. The NFs were prepared using probe-type sonication in a water–ice bath.

### 2.4. Metal Oxide Nanoparticles

Metal oxides, including copper oxide, zinc oxide, titanium dioxide, and iron oxide, can absorb solar radiation with limitations imposed by their bandgap. Upon light absorption, electrons are excited from the valence band to the conduction band, generating electron-hole pairs [71]. The relaxation from excited states releases energy in the form of heat [72]. The efficiency of light-to-heat conversion in metal oxides is highly dependent on factors such as material composition, crystal structure, and surface properties.

Copper oxide NPs have emerged as a prominent material in solar thermal conversion research due to their unique properties. In a recent study conducted by Moghaieb et al. [29], the authors synthesized surfactant-free CuO_x_ NPs by plasma-induced nonequilibrium electrochemistry, which featured a sacrificial copper foil anode and a capillary tube cathode. The researchers prepared a 6 mL electrolyte solution by combining equal parts of sodium chloride, sodium nitrate, and sodium hydroxide, without the addition of reducing, capping, or stabilizing agents. They maintained atmospheric-pressure microplasma for 30 min at the electrolyte surface, leading to a visible colour change from transparent to orange. Following centrifugation, heat drying, and annealing at 400 °C for 6 h, a completely black powder was obtained, suggesting a phase transformation due to thermal oxidation. Both annealed and nonannealed CuO_x_ NP powders were then separately re-dispersed in ethylene glycol (EG) at different volume fractions and subjected to sonication to achieve homogenous dispersions. In an extensive study by Milanese et al. [73], the researchers investigated a broad array of metal oxide water-based NFs, encompassing commercially available Al_2_O_3_, CuO, and TiO_2_, as well as CeO_2_, ZnO, and Fe_2_O_3_, prepared via the hydrolytic synthesis method. For instance, the CeO_2_ NPs were synthesized by dissolving 2.6 mmol Ce(NO_3_)_3_·6H_2_O in methanol, followed by the addition of acetylacetone. Subsequently, a 30 wt% ammonia solution in water was introduced to the mixture, which was then vigorously stirred for 24 h. The final suspensions of CeO_2_, ZnO, and Fe_2_O_3_ NPs were acquired after centrifugation, ensuring optimal particle collection. Moreover, magnetic metal oxides have gained considerable interest due to their unique magnetic properties. In a study by Balakin et al. [74], the authors prepared a magnetic Fe_2_O_3_/water NF by mechanically stirring Fe_2_O_3_ NPs in distilled water and subjecting the suspension to bath-type sonication at 130 W for 30 min. They refrained from using surfactants to avoid potential electromagnetic and rheological impacts on the solar thermal conversion process. In a related study, Hosseini and Dehaj [75] prepared Fe_2_O_3_/water and Fe_3_O_4_/water NFs by employing more powerful sonication (probe ultrasonicator at 300 W) and incorporating the surfactant sodium dodecylbenzene sulfonate (SDBS) with a neutral pH to improve colloidal behaviour. Afterwards, the mixture was re-sonicated using a bath-type sonicator. They ultimately obtained two NFs at volume fractions of 0.005%, 0.01%, and 0.02%, further expanding the range of potential applications in solar thermal conversion processes.

Considering the plasmonic absorption properties of Ag NPs and antimony-doped tin oxide (ATO) NPs in the visible and near-infrared spectra, Sreekumar et al. [76] developed a multicomponent ATO–Ag/water NF to enable broader absorption across these regions and potentially enhance STC efficiency. They prepared the mixture through a reduction reaction involving Sb_2_O_5_, SnCl_2_, HCl, AgNO_3_, and ATO NPs. Following the chemical reduction process, the ATO–Ag NP solution was washed and dried in a hot air oven at 60 °C. The ATO–Ag NPs were finally re-dispersed in distilled water and sodium dodecyl sulfate (SDS) surfactant was added to improve the stability of the NFs. As an example of a semiconducting nitride, titanium nitride (TiN), which exhibits strong plasmonic absorption, can be highlighted. Wang et al. [77] prepared TiN NPs/EG by dispersing 0.1135 g of TiN NPs in 1000 mL of EG, followed by simultaneous stirring and ultrasonication for 30 min, resulting in a concentration of 0.01 wt.%. Further dilutions using ultrasonication yielded concentrations of 0.001 wt.%, 0.003 wt.%, 0.005 wt.%, and 0.007 wt.%.

**Table 1:** The synthesis and morphology of various types of nanomaterials and the preparation, optical properties, and stability of their nanofluids.

Nanofluid	NP Synthesis Method	NP Morphology	NP Loading	Stabilizer	NF Preparation	Measured Optical Property	Stability Tests	[Ref] Year
- Au NPs/EG - CuO QDs/EG - Au NPs–CuO - QDs/EG	Plasma-induced nonequilibrium electrochemistry (PiNE) synthesis at atmospheric pressure	- Different shapes of 27 nm mean diameter - Nearly spherical of 3.3 nm mean diameter	- 0.0055 vol% - 0.0017 vol%	Electrostatic stabilization	Heat drying of the as-prepared samples followed by sonication in EG	Absorption and scattering	11 weeks of ambient storage	[61] 2020
Au NPs/water	Seed-mediated synthesis method	Average sizes of 25 nm, 33 nm, and 40 nm	0.07 mg/L, 0.18 mg/L, and 0.39 mg/L	CTAC surfactant and ultrasonication (30 min at 20 °C)	Ultrasonication for 30 min at 20 °C	Absorbance	16 h of ambient storage and heating at 90 °C	[58] 2016
Au NPs/water	Citrate, PEG, PVP, and BSA coatings	-	0.1611 mg/mL	PEG, PVP, and BSA dispersants	The excessive polymer was filtered, and samples were diluted	-	3 years of ambient storage, continuous irradiation, and cyclic irradiation	[60] 2021
TiN/EG	Commercial	30 nm diameter	0.01, 0.001, 0.003, 0.005, and 0.007 wt.%	-	30 min sonication and dilution	-	-	[43] 2021
ATO–Ag/water	Chemical reduction	Nonspherical of an average size <40 nm	0.01–0.2 wt%	-	Dispersion in distilled water by adding SDS surfactant	Extinction	-	[76] 2020
- Ag NPs/water - SiO_2_ NPs/water - Ag NPs@SiO_2_/water	A reducing–oxidizing method with adding citrate followed by Coating Ag NPs with SiO_2_ using TEOS	- 38–44 nm diameter - 141 nm diameter	6 mL	- TEOS shell - PVP dispersant	- Dilution	-	3 weeks of ambient storage and solar radiation (simulated and real)	[62] 2020
- Al_2_O_3_/water - CuO/water - TiO_2_/water - ZnO/water - CeO_2_/water - Fe_2_O_3_/water	- Commercial (Al_2_O_3_, CuO and TiO_2_) - Hydrolytic synthesis (ZnO, CeO_2_, and Fe_2_O_3_)	-	0.05–1 vol%.	-	Direct dispersion and centrifuge	Extinction	-	[73] 2016
- Fe_2_O_3_/water	Commercial	60 nm (110 nm after heating)	0.5–2 wt%	-	Mechanical stirring and sonication for 30 min at 130 W	-	-	[74] 2022
- Fe_2_O_3_/water - Fe_3_O_4_/water	Commercial	20–50 nm diameter	0.001, 0.01, and 0.02 vol%	SDBS dispersant	Ultrasonication for 20–60 min at 300 W	-	-	[75] 2022
- CQDs/PEG-200	- Microwave heating method - 5 mL	Less than 10 nm dimeter	-	PEG-200	Stirring of 22 mL (∼500 rpm)	-	One month of ambient storage	[68] 2022
- GO/[BMIM]BF_4_ - MWCN/[BMIM]BF_4_ - Ti_3_C_2_/[BMIM]BF_4_	- Commercial (GO and MWCN) - Solvent etching (Ti_3_C_2_)	- 0.5–5 μm diameter - 3–5 nm and 8–15 nm inner and outer diameters	- 0.001, 0.002, 0.005, 0.02, and 0.04 wt% - 0.02 wt% - 0.02 wt%	-	- Ultrasonication for 7 min (400 W, 20 kHz)	-	-	[69] 2022
- SWCNTs/water, /PG, or /therminol-55 - MWCNTs/ water, /PG, or /therminol-55	Each material was chemically functionalized by acid and potassium persulfate (KPS)	-	-	- Oxygenated, carboxyl, and hydroxyl groups - SDBS dispersant	- Ultrasonication at 20 W in a water–ice bath for 20 min	-	Working temperature change (80–250 °C)	[70] 2016
- CIT–Au NPs/water - PEG–Au NPs/water	- Citrate reduction by the Turkevich method	Near-spherical particles of 10 nm diameter	- 0.0358–0.358 mg/mL	- CIT coating - PEG coating	The as-made samples were diluted after post-synthesis purification	- Extinction	16 months of ambient storage and heating for 12 h	[78] 2019
- MWCNT/water - MWCNT–TiN/water	Commercial	5–10 nm and 20–30 nm inner and outer diameters and 10–30 μm length - TiN of 20 nm diameter	10, 20, and 30 ppm	CTAB surfactant	Magnetic stirring at 800 r/min for 30 min - Sonication for 60 min at 750 W and 20 kHz	-	One week of ambient storage	[79] 2022
- Ag/water - Fe_3_O_4_/water - Graphite/water	Commercial	- 50 nm - 15 nm - 20 nm	-	TPABr surfactant and sulfuric acid treatment	-	-	-	[80] 2016
SiC–MWCNTs/EG	-	- SiC: 40 nm in diameter - MWCNT: 20 nm diameter and 30 μm length	0.01–1 wt%	PVP–K30 and hexane dispersant and ultrasonication for 1 h	-	-	One month of ambient storage, mass fraction, temperature, and thermal cycling	[81] 2020
- Graphene/water	Commercial	Nanoplates of 2 nm thickness and 2 µm diameter	0.00025–0.005 wt%	-	-	Extinction	-	[82] 2016
- CeO_2_/Chloroform - Fe_2_O_3_/Chloroform	-	- Spherical (3.0 ± 0.4 nm) - Spherical (4.3 ± 1.9 nm)	- 3 mg/mL - 5 mg/mL	-	-	Absorbance	-	[73] 2016
- Ag/water - Fe_3_O_4_/water - Graphite/water	-	- Commercial - Mean diameter is 50 (Ag), 15 (Fe_3_O_4_), and 20 (graphite)	40 ppm	-	-	Extinction	-	[80] 2016
- Ag NPs/water - Ag NPs@SiO2/water	-	- 41 nm diameter - 50–100 nm diameter	-	TEOS shell, Na_2_SiO_3_ shell, and citrate coating	-	-	Cyclic heating and cyclic UV radiation	[83] 2018
- Al_2_O_3_/water - TiO_2_/water	-	- 13 nm - ~21 nm	0.1 and 0.3 vol%	-	-	Absorption and scattering	-	[84] 2014
- CuO/water–EG - Al_2_O_3_/water–EG - CuO–Al_2_O_3_/water–EG	-	- Spherical of 40 nm average size - Spherical of ~100 nm average size	- 0.0001–0.0003 vol% - 0.004–0.008 vol%	SHMP surfactant, pH control, and ultrasonication	-	Extinction	Two weeks of ambient storage	[85] 2016
ZrC/water	-	40 nm diameter	-	Ultrasonication for 10 min)	-	-	-	[86] 2019
- GE/water - GO/water - RGO/water	-	GO: nanosheets of 0.55–1.2 nm thickness and 0.5–3 µm size	-	PVP	-	-	Two months of ambient storage	[87] 2017
- GO/EG–water - rGO/EG–water - rGO/water	-	-	-	- UV exposure - PVP dispersant	-	-	Two months of ambient storage	[88] 2019
- MWCNTs/water	-	20 nm diameter and 1–25 µm length	-	SDBS, CTAB, SDS, and Triton X-100	-	-	- One month of ambient storage	[89] 2018
- BN/EG - BN–CB–/EG	-	-	- 30–90 - 4–15 (ppm)	-	-	-	-	[90] 2021
- CuO/EG	- Plasma-induced nonequilibrium electrochemistry (PiNE)	- flower-like morphology of sizes up to 200 nm	- 0.001–0.01 vol%	- No surfactant used	- Ultrasonication in EG	- Absorption and scattering	- 30 days of ambient storage - Cyclic heating	[29] 2023
- Au NPs/Water - Au NPs/ Azadirachta Indica–water	- Turkevich method	- 30 nm	- 4 mg/L	- PVP	-	- Absorbance	- 30 days of ambient storage	[59] 2023
- C NPs/Water-PSS	- Modified vortex trap method (VTM)	- amorphous carbon, graphene oxide, and reduced graphene oxide with a mean particle size of 30 nm	- 0.025–0.5 wt%	-	-	-	- Ambient storage	[66] 2023

## 3. Energy Flow in Solar Thermal Nanofluids Used in DASCs

**Figure 3:**
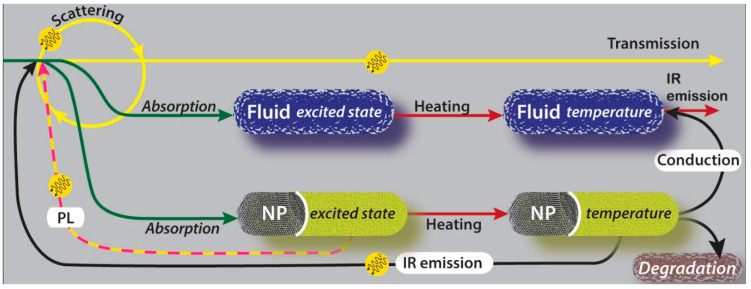
Energy flow in solar thermal nanofluids (nanoparticles and base fluid).

Light interaction with an NF can give rise to several physical and chemical phenomena (Figure 3), establishing a flow pattern for the energy originating from solar photons. Here we consider phenomena taking place within the NF, as interface interactions such as reflection, etc., at the front- and back-end of the NF can be discussed under textbook optics. The extent of energy exchange and the mechanisms involved strongly depends on the material characteristics of the NP in the NFs [91,92,93,94,95,96] as well as the size/shape of the NPs and their concentration in the NF. With reference to Figure 3, heating of the working fluid, hence its temperature increase, is ultimately the desired ‘finish-line’ for the energy flow. Note that the temperature of the NPs may not be at equilibrium with the fluid temperature under solar irradiation, hence the NPs and the working fluid should be considered separately.

Transmission, scattering, and absorption are the three main light–matter interaction events to be considered. As illustrated in Figure 3, the two components of NFs, i.e., the NPs and the base fluid, interact differently with light. While both can absorb light to varying degrees with different patterns over the solar spectrum, only NPs can scatter light. Transmitted light is lost energy with no possibility of being recovered. Light that is scattered when travelling through the NF [96] can be in part ‘recycled’ with chances of being completely lost through transmission, scattered again, or eventually absorbed. Hence, scattering can only lead to partial light re-absorption and should be minimized in favour of direct absorption. Absorbed light, either by the working fluid or the NPs, is energy that becomes available for conversion into heat; however, several energy-loss paths are still possible, especially after absorption by the NPs.

Direct light absorption by the working fluid is a very efficient energy conversion process as the only loss mechanism after absorption is represented by the fluid IR emission if the NF is adequately insulated. NP light absorption initially produces an excited state for electrons and the subsequent decay is highly dependent upon the material characteristics and composition. For instance, metallic NPs generally exhibit fast phonon emissions [97,98], hence increasing the NP temperature very quickly before any other loss mechanism can take place. Some metals or materials with high carrier concentrations can exhibit plasmonic absorption, i.e., enhanced absorption at specific wavelength ranges. This can be beneficial if the absorption range is within the visible and still relatively broad. However, plasmonic electronic excitation is often abrupt and generates high electric fields at the NP surface, which could result in cavitation (in extreme cases) or sudden temperature changes at the NP–fluid interface that could be detrimental to the chemical stability of the NFs.

In nonmetallic NPs that feature an energy bandgap, such as metal oxide or semiconducting NPs, electrons decay through phonon emissions down to the bandgap edge; there, phonon emission is either prohibited or sufficiently slow for other mechanisms to take place. A similar process can take place due to surface states, where electron relaxation through phonon emission can be prevented. For instance, photoluminescence (PL), i.e., the re-emission of a photon of a lower wavelength than the one that was absorbed, can occur in these cases. Similar to scattering, PL can recycle the light, and minimizing this phenomenon in the UV–Vis range in favour of direct absorption is preferred. Slow electron decay, especially due to surface states can also enhance NP–fluid chemical reactions and possibly pose a challenge to NF stability through its degradation. Hence fast phonon emission is the preferred channel also for metal oxide and semiconducting NPs, where a higher bandgap generally reduces heating and increases energy losses.

Upon heating the NP and a temperature rise, heat conduction from the NPs to the working fluid takes place. Stabilizers or surfactants that are often used in chemical synthesis introduce an additional thermal resistance which could locally increase the temperature at the interface between the NPs and the working fluid. Localized heating due to surfactants or via plasmonic absorption may have an impact on the chemical stability of the NFs and can represent a form of energy loss that has received little attention so far. Localized heating can result in the degradation of NPs and agglomeration over time under several operating conditions such as high solar and thermal loadings as well as solar and thermal cycling. This type of energy loss to chemical energy (transformation of matter) is extremely important not only because it reduces the STC efficiency but also because it can impact the chemical/physical stability of the NFs, hence their long-term applicability.

As NPs exhibit temperature values that are higher than the working fluid, heat conduction occurs only in one direction, i.e., from the NPs to the working fluid. While NP IR emissions can be significant in some cases, this can be often re-absorbed directly by the working fluid and should not represent a major loss mechanism.

## 4. Fundamental Optical Characterisation of Nanofluids for DASCs

**Figure 4:**
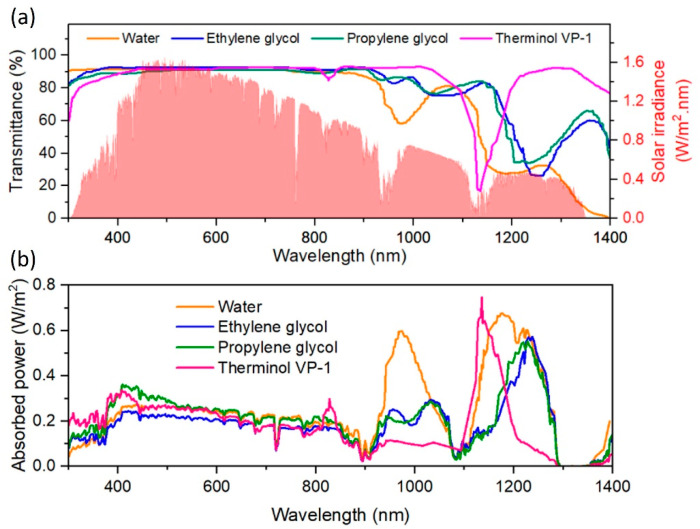
(**a**) Spectral solar irradiance at Earth’s level (air mass of 1.5, AM1.5) [102] and (**b**) transmittance of common base fluids used in DASCs for a one cm sample thickness (transmittance for Terminol VP-1 is taken from [29]).

Conventional base fluids used in DASCs are strong absorbers in the UV and IR regions, which represents 57% of the total solar energy; however, they are transparent in the Vis part, meaning that 43% is mostly lost as transmitted light [20]. Figure 4a reports the solar irradiance together with the transmittance spectra for four relevant working fluids used in DASCs. The optical characteristics of the working fluids result in power absorbed (Figure 4b), which clearly shows that very little solar irradiance is used up to ~900 nm. The main functionality in adding NPs to these base fluids is, therefore, to compensate for this by absorbing the solar energy present in the Vis region. In this view, studying the absorption and scattering behaviours of NFs is essential in evaluating their potential as working media in DASCs since this determines to what extent the NF can effectively utilize solar radiation energy [99,100]. For the performance improvement of DASCs, it is ultimately desirable to increase absorption, which can be impacted by the shape, size, material type, and concentration of the NPs, as well as the type of base fluid [101].

Experimentally, when taking measurements in the lab, the light interaction with an NF sample is characterized by the transmittance ($T\left(\lambda \right)=I_{tr}\left(\lambda \right)/I_{inc}\left(\lambda \right)$), absorptance ($Abs\left(\lambda \right)=I_{abs}\left(\lambda \right)/I_{inc}\left(\lambda \right)$), and scattering ($S\left(\lambda \right)=I_{sct}\left(\lambda \right)/I_{inc}\left(\lambda \right)$), which correspond to fractions, with values from 0 to 1, of the incident light intensity ($I_{inc}\left(\lambda \right)$), hence

(1) T(λ)+Abs(λ)+S(λ)=1

All the optical quantities are wavelength-dependent (λ); however, here below we have omitted this dependence for simplicity. Furthermore, the interaction of light with an NF sample is three-dimensional in nature; however, a one-dimensional analysis is applicable for the measurements of the fundamental physical NF properties where the sample size can be minimized in two of the three dimensions and ensuring re-absorption is negligible. The light that is not transmitted (i.e., *Abs* + *S* or 1−*T*) is also referred to as attenuated light. From a practical point of view, both scattered and transmitted light represents the light that has escaped the sample, the latter without any change in the direction of the propagation. However, experimental measurements of *S* and *T* include more than purely scattering and transmission phenomena and their values result from the sum of all of the light exiting the sample. If, for example, samples exhibit strong PL or IR emissions (Figure 3), the corresponding light emission is measured as either scattering or transmission. As previously discussed, PL and IR emissions should be generally considered losses to be minimized and should be expected to be negligible. Optical measurements of NF samples then allow the extraction of fundamental physical NF properties such as extinction ($\mu_{ext}$), absorption ($\mu_{abs}$), and scattering ($\mu_{sct}$) coefficients measured in m^−1^, which can then be used, for instance, to simulate full DASC system performance in three dimensions [29].

**Figure 5:**
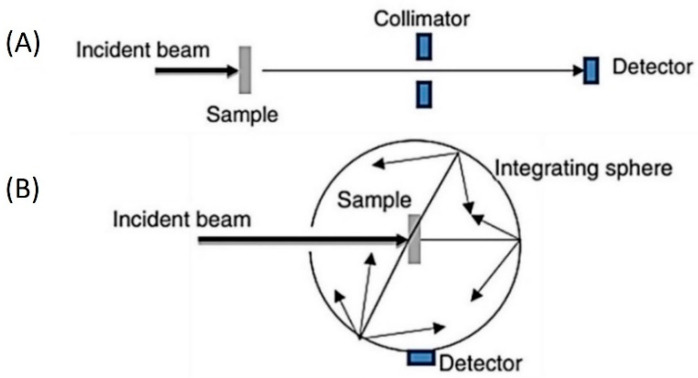
Schematic shows methods of direct measurements of (**A**) transmittance using the transmission port and (**B**) transmittance and scattering combined in the integrating sphere compartment [106].

The total attenuation of light while travelling through an NF sample with a given depth (or thickness, *L*) can be evaluated by measuring the transmittance using a spectrophotometer. Most conventional spectrophotometers can cover electromagnetic spectral ranges between 300 nm and 900 nm, including the ultraviolet and visible regions, as well as part of the near-infrared, and this can extend into higher wavelengths depending on the type of the instrument used, e.g., up to 3000 nm. The transmittance is determined by the ratio of the light intensity transmitted through the NF sample ($I_{tr}$), and collected by the detector to the intensity of the incident light, i.e., the intensity of the light source incident on the sample and prior to interacting with the sample ($I_{inc}$), see Figure 5A. The latter needs to be measured with an appropriate reference sample, which can take into account instrumental losses including those due to the cuvette absorption and reflection at the interfaces. As most working fluids have negligible attenuation in relevant UV and Vis ranges, in order to evaluate the impact of NFs, it is convenient to use a reference with just the working fluid (i.e., without NPs). This will, however, limit the validity of the NF optical properties to these ranges where the working fluid has little interaction with the light (e.g., <900 nm). In order to further extend the validity of the measurements to the IR, more complex calculations and verifications would need to be introduced.

The extinction coefficient of the NF can be calculated from the Beer–Lambert law [96]:

(2) $$T\left(L\right)=\frac{I_{tr}}{I_{inc}}=e^{-\mu_{ext}L}$$

(3) $$\mu_{ext}=-\frac{lnT}{L}$$

Attenuation is due to both absorption as well as scattering, hence the extinction coefficient is the sum of the corresponding coefficients:

(4) $$\mu_{ext}=\mu_{abs}+\mu_{sct}$$

The contribution of scattering can be neglected in some conditions, e.g., for NPs with sizes much smaller than the incident wavelength [103,104,105] and at low particle concentrations with no agglomeration (<0.01% volume fraction) [104]. In this case, the extinction coefficient can be representative of the absorption coefficient ($\mu_{ext}\approx \mu_{abs}$) [96]. Larger particles, high particle concentrations and/or the presence of agglomeration can lead to significant scattering, which requires the spectrophotometer instrument to be equipped with an integrating sphere system (Figure 5B) to yield transmittance and scattering separately. Depending on the spectrometer and integrating sphere, separate measurements of *T* and *S* may not be possible, so scattering is calculated from the measurements of *T* and (*T* + *S*), by difference. In general, it is difficult to have a priori knowledge of the light–NF interactions and the general case where both absorption and scattering coefficients are not negligible should be assumed.

The light absorbed (*I_abs_*) by an NF sample of depth *L* can be described in terms of the transmitted light intensity *I_tr_* at depth x, multiplied by the absorption coefficient, and where Equation (2) has been used:

(5) $$I_{abs}=\int_{0}^{L}\mu_{abs}I_{tr}\left(x\right)\cdot dx=\mu_{abs}I_{inc}\int_{0}^{L}e^{-\mu_{ext}x}\cdot dx=\mu_{abs}I_{inc}\left(\frac{1-e^{-\mu_{ext}L}}{\mu_{ext}}\right)=\mu_{abs}\frac{I_{inc}\left(1-T\right)}{\mu_{ext}}$$

The absorption coefficient can be then determined by rearranging Equation (5) and substituting the values produced by the measurements of *T* and (T+S) as follows:

(6) $$\mu_{abs}=I_{abs}\frac{\mu_{ext}}{I_{inc}\left(1-T\right)}=\frac{I_{abs}}{I_{inc}}\frac{\mu_{ext}}{\left(1-T\right)}=\left[1-\left(S+T\right)\right]\frac{\mu_{ext}}{\left(1-T\right)}=-\frac{\left[1-\left(S+T\right)\right]}{\left(1-T\right)}\frac{lnT}{L}$$

Consequently, the scattering coefficient can be simply determined by subtracting the absorption coefficient in Equation (6) from the extinction coefficient in Equation (4).

(7) $$\mu_{sct}=-\frac{lnT}{L}-\left\{-\frac{\left[1-\left(S+T\right)\right]}{\left(1-T\right)}\frac{lnT}{L}\right\}=\frac{lnT}{L}\left\{\frac{\left[1-\left(S+T\right)\right]}{\left(1-T\right)}-1\right\}$$

**Figure 6:**
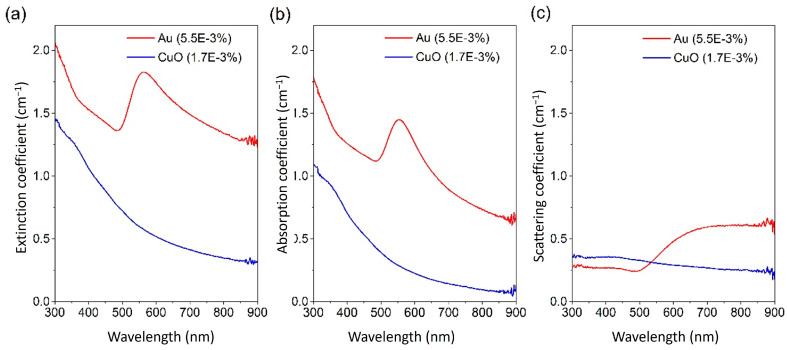
(**a**) Extinction, (**b**) absorption, and (**c**) scattering coefficients for gold (red) and copper oxide (blue) NPs in an ethylene glycol base fluid. Adapted with permission from [61].

This approach was used, for instance, to evaluate the properties of surfactant-free Au NPs prepared by McGlynn et al. [61], which exhibited a variety of shapes and sizes, with most particles consisting of small spherical NPs with an average diameter of 27 nm. They evaluated the absorption and scattering behaviours of their NF (Au NPs in ethylene glycol) across the wavelength range of 300–900 nm, and the results revealed that both absorption and scattering coefficients increased with the NP concentrations, as expected. In addition, the absorption coefficient is dominated by the plasmonic absorption between 540 nm and 560 nm (red in Figure 6b), which is much higher than the corresponding scattering coefficient (red in Figure 6c) for most of the spectral range.

**Figure 7:**
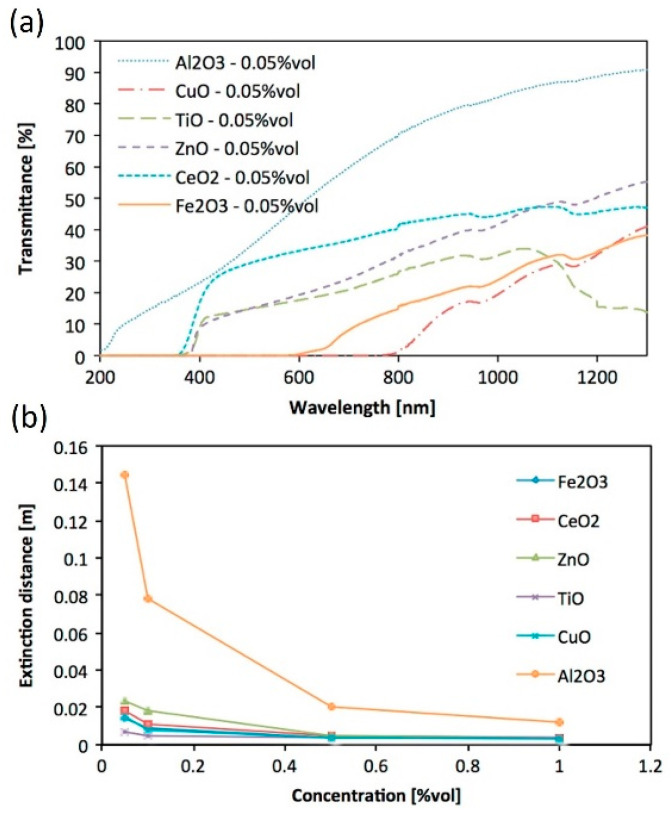
(**a**) Comparison between the transmittance of several NFs as a function of wavelength. (**b**) The extinction distance for investigated NFs as a function of NP concentration, in the case of wavelength equal to 1300 nm. Adapted from [73].

Many reports use absorbance to characterize NFs; however, this parameter does not allow for a direct comparison with other NFs and cannot be used directly in simulations [58,78]. The transmittance of the water-based metal oxide NFs of Al_2_O_3_, CuO, TiO_2_, ZnO, CeO_2_, and Fe_2_O_3_ prepared by Milanese et al. [73] at 0.05–1 vol% were measured over 200–1300 nm using a 3 mm path-length quartz cuvette. As depicted in Figure 7a, at 0.05 vol%, the six materials exhibited three transmittance patterns where CuO and Fe_2_O_3_ transmitted light only in the near-IR region at wavelengths above 800 nm and 600 nm, respectively, while TiO_2_, ZnO, and CeO_2_ transmitted more in the Vis and near-IR region at wavelengths above ~380 nm; the last material, Al_2_O_3_, had the lowest performance with most of the light transmitted. In Figure 7b, the distance at which all incident light was attenuated was calculated at different concentrations for the six materials and showed that Al_2_O_3_ is the worst material while the other five materials have much higher attenuation rates within short distances of less than 0.02 m at a concentration as low as 0.05 vol%. The extinction coefficient of ATO–Ag NPs [76] with an average particle size of 40 nm and dispersed in water at 0.01–0.2 wt% was calculated over 300–900 nm, and its peak was 8.2 cm^−1^ at 300 nm at 0.2 wt% and dropped to 3.7 cm^−1^ at 900 nm. It was also observed that the extinction coefficient exhibited approximately a linear function with the mass fraction. By dispersing graphene nanoplates with a thickness of 2 nm and diameter of less than 2 µm in deionized water at 0.00025–0.005 wt% [82], the NFs demonstrated a strong absorption band in the range of 250–300 nm, with complete absorption across the range of 1400–1550 and above 1850 nm. In addition, the NF with the highest concentration of 0.005 wt% exhibited the highest extinction coefficient with a peak of 6.5 cm^−1^ at 300 nm, which decreased gradually to below 4.5 cm^−1^ at wavelengths above 700 nm. Milanese et al. [73] studied the effect of temperature change within a range of 100–500 °C in 100 °C steps on the optical properties of ZnO, CeO_2_ (~4.3 nm in diameter), and Fe_2_O_3_ (~3.0 nm in diameter) in chloroform solution at 3 mg/mL and 5 mg/mL for Fe_2_O_3_ and CeO_2_ NPs, respectively.

**Figure 8:**
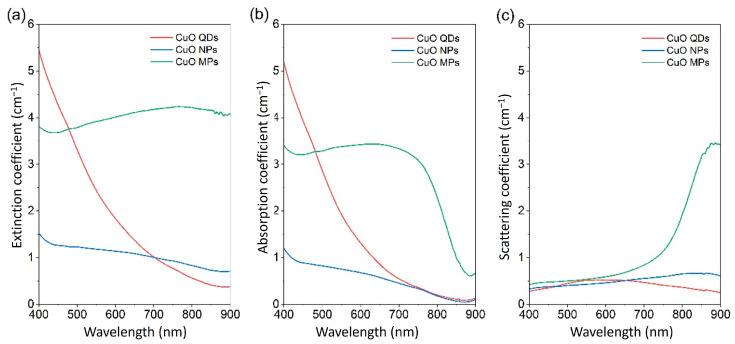
Coefficients of (**a**) extinction, (**b**) absorption, and (**c**) scattering of three different types of CuO at different sizes dispersed in ethylene glycol (mean diameter: ~3.5 nm QDs, ~50 nm NPs, ~3–10 µm MPs).

Similar to absorbance, the use of attenuation or extinction coefficients, or transmission only, cannot differentiate between absorbed and scattered light, hence the results can be misinterpreted. In general, assessing the optical properties of NFs using transmittance or absorbance or extinction coefficient only can be misleading. For instance, we have produced NFs from three different types of CuO particles. We have used quantum dots (QDs) [107], commercially available NPs and microparticles (MPs) (Sigma Aldrich with CAS numbers of 1317-38-0 and 1317-38-0, respectively), and each sample of the three CuO particles was dispersed in 10 mL of ethylene glycol (Sigma, CAS number 107-21-1) at a volume fraction of 0.005%. The re-dispersion process was performed for 1 h in a bath-type sonicator (VWR USC300TH, 230 V, 50 Hz, 370 VA) and then for 3 min in a probe-type sonicator (VCX-130PB ultrasonic processor, Sonics Materials) at 80% (104 W) for high dispersion homogeneity. The impact of the different sizes on the scattering is immediately clear in Figure 8c.

In this case, using the extinction coefficient to characterize the optical behaviour of QDs (red in Figure 8a) is a suitable approach for the part of the wavelength range under analysis as scattering (red in Figure 8c) is very low in comparison to the absorption coefficient (red in Figure 8b). However, the scattering coefficient of NPs (blue in Figure 8c) is comparable to the absorption coefficient (blue in Figure 8b), hence the first cannot be neglected. This is even more obvious for MPs where the extinction coefficient (green in Figure 8a) is very high throughout the full wavelength range. However, the analysis of absorption and scattering separately (green in Figure 8b,c, respectively) shows that much of the extinction is due to scattering in the range 800–900 nm. In this specific case, the higher scattering is most likely all due to the relationship between incident wavelength and particle size as per Mie’s theory. However, we should note that scattering can take over the absorption also due to other light–NP interaction phenomena, such as is the case for NPs with plasmonic properties (e.g., red in Figure 6c) as plasmon resonance enhances scattering above the plasmonic peak wavelength. It is therefore important to evaluate NF properties appropriately for these to be used in assessing NF performance and in more complex analytical and simulation efforts.

## 5. Characterization of the Stability of Nanofluids for DASC Applications

Nanofluids are colloids of nanoparticles within base fluids and it is critical for the performance of NF-based DASCs to address the long-term stability under various thermal and solar radiation operating conditions. Dispersed particles are prone to destabilization under the effect of forces, such as Van der Waal and gravity, which can induce agglomeration leading to the formation of larger particles/agglomerates and enhancing sedimentation. Other forces, such as buoyancy, steric, and electrostatic forces, can play opposite roles in improving particle stability, and nanofluid stability is dependent on which group of forces dominates. The formation of large agglomerates leads to a sequence of operational issues and contributes to the deterioration of the thermal and optical properties of NFs, affecting the overall performance of DASCs. Such destabilization/stabilization forces are highly affected by the operating conditions (temperatures, particle loading, and incident solar radiation). In addition, the chemical stability of NFs and the NPs or working fluid separately is important to maintain NF properties across operational conditions.

High operating temperatures increase Brownian motion leading to higher particle collision frequencies, also enhanced by a reduction in viscosity of the base fluid at higher temperatures. Temperature change also has an impact on the surface charge and zeta potential of the particles [108]. In addition, an increase in temperature can lead to the degradation of surfactants in the case of steric-stabilized NPs [109]. Direct exposure of the NFs to concentrated radiation (intensity higher than one sun) can cause the chemical deterioration of surfactants and functional groups and even of the chemical composition or phase of the NPs, particularly from UV radiation [110]. Furthermore, medium- and high-temperature DASCs operate at high solar radiation intensity, which requires relatively high particle loadings, i.e., more particles per unit volume and consequently shorter interparticle distance, which induce agglomeration of particles at higher rates compared to low particle loadings. Moreover, due to heating during exposure to sunlight in the daytime and cooling during nighttime, this daily cyclic heating/cooling impacts several parameters, including changes in the morphology of the NPs that could promote agglomeration and chemical reactions such as oxidation [83,111] as well as a reduction in thermophysical properties [112]. For these reasons, the chemical stability of NPs needs to be assessed in the specific working fluid as several chemical reactions could be taking place at the NP–fluid solid–liquid interface that would not normally take place in other environmental conditions.

The stability of NFs can be assessed following various methodologies, which can produce operational stability parameters and an understanding of relevant underlying mechanisms. The observation of discolouration and/or particle sedimentation of the NFs is perhaps one of the simplest and most direct assessment methods. However, seldom can it provide quantifiable operational measures and scientific insights. On the other hand, measuring the changes in the optical properties, morphology, surface chemical composition, etc., of the NPs can be more helpful in analysing and understanding stability.

Sharaf et al. [78] extensively investigated the stability of polymer-coated Au NPs/water against several destabilization factors, including ambient storage for 16 months, elevated temperature, continuous and cyclic modes of solar radiation, and intensified UV radiation. In their work, they tested four samples: CIT–Au NPs and CIT–Au NPs coated using three different polymers to give BSA–AuNPs, PVP–AuNPs, and PEG–AuNPs. The results revealed that the ambient dark storage for three years did not affect the stability and PEG–AuNPs demonstrated the best long-term stability. The absorbance measurements showed that no plasmonic wavelength shifts or shoulder evolutions took place in all four NFs. The absorbance peak intensity, however, increased for PVP– and BSA–Au NPs by 9.0% and 9.5%, and this was attributed to changes in the thickness of the adsorbed polymer coating over time, while the peak intensity of CIT–Au NPs dropped by 9.6% due to sedimentation. In addition, after the samples were exposed to broadband solar radiation at different intensities for 12 h, CIT–Au NPs resulted to be the most stable with no changes in absorbance intensity, while both PEG– and PVP–Au NPs showed a drop in absorbance and the hydrodynamic diameter under radiation exposure. However, no shifting or broadening in the absorbance peaks of PEG– and PVP–Au NPs were observed, indicating that they experienced no agglomeration after being exposed to radiation. Furthermore, cyclic radiation for 20 cycles at one-sun intensity for 8 h followed by 16 h in the dark produced more significant changes in absorbance and hydrodynamic diameter compared to exposure to continuous radiation, with the CIT–Au NPs less impacted than the polymer-coated samples. Moreover, they tested the NFs at elevated temperatures of 55 °C, 70 °C, and 85 °C for 12 h, and all NFs showed excellent thermal stability confirmed by their absorbance measurements. The stability of the same NFs (aqueous NFs with Au NPs coated by CIT, PEG, PVP, and BSA) was then investigated for a much longer period [60]. Over three years of ambient storage, continuous irradiation at varying intensity for 12 h and 20 consecutive cycles of one-sun illumination (8 h under light, followed by 16 h in the dark), the NFs exhibited no shifts in their plasmonic absorption peak wavelength and insignificant changes in the hydrodynamic diameter. They also reported that CIT-coated AuNPs were the most stable under continuous solar radiation. The effect of ambient storage on the stability of CTAC surfactant-based Au NPs/water was investigated by Chen et al. [58] and they found that the NFs were stable after days but the increase in temperature up to 90 °C worsened the stability and rapid sedimentation appeared several hours after the experiments. They attributed the post-experiment instability to agglomeration induced by Brownian motion and surfactant degradation at high temperatures.

**Figure 9:**
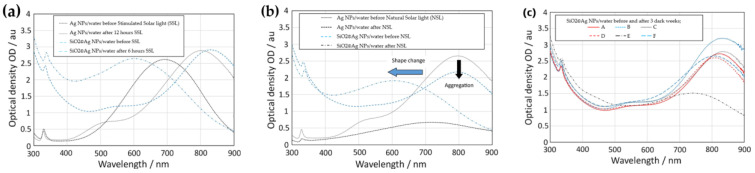
UV–Vis spectra of Ag NPs and SiO2@Ag NPs in water: (**a**) after exposure to stimulated solar light, (**b**) after exposure to natural solar light for two weeks, and (**c**) after storage in the dark for three weeks (adapted from [62]).

Gorji and Ranjbar [80] measured the zeta potential of three different types of materials (Ag NPs, Fe_3_O_4_ NPs, and graphite) in water and found that the graphite/water exhibited the highest value of 45.1 mV compared to 41.3 mV and 38.7 mV for Fe_3_O_4_/water and Ag NPs/water, respectively, all at a pH of 9.5–10. Coating Ag NPs with SiO_2_ and then dispersing them in water to form Ag NPs@SiO_2_/water NFs resulted in better stability when compared with uncoated Ag NPs in the same base fluid, which exhibited poor stability under both heating and UV radiation cycles [83]. Kimpton et al. [62] investigated the stability of both Ag NPs/water with and without coating with SiO_2_. They monitored the stability of both NFs over three weeks of ambient storage, as well as under exposure for a period of two weeks to simulated and natural solar radiation, the latter during the first half of June 2018 in the UK. As shown in Figure 9, they assessed the impacts of destabilization factors by studying the UV–Vis spectra and the morphology of the particles via transmission electron microscopy (TEM). Their results revealed a lot of interesting points when comparing bare Ag NPs and SiO_2_-coated Ag NPs, as well as differences due to the use of simulated and natural light. For instance, SiO_2_-coated Ag NPs showed less stability than the uncoated Ag NPs, as the former showed a higher reduction and position shifting in the absorption peak after ambient dark storage and upon exposure to simulated light. This lack of stability was explained by the changes in the morphology of the SiO2@Ag NPs samples verified by TEM. They found a reduction in the size of Ag NPs inside SiO_2_ shells by about 10 nm suggesting that the silver was being etched inside the coating. In addition, they found degradation of the SiO_2_ shell, since it had reduced in size, and some Ag NPs were found no longer coated. Based on these results, they carried out further modifications by using thicker SiO_2_ coatings to improve stability. However, they found that increasing the coating thickness accelerated the change in morphology of Ag NPs under exposure to light. On the other side, exposure to natural sunlight revealed similar results to that obtained under exposure to simulated light in terms of the shift of the absorbance peak position, but different in the reduction in the peak values. They attributed this to a faster change in the morphology of the particles.

Zheng et al. [79] studied the dispersion stability of CTAB surfactant-based NFs of CNT/water and CNT–TiN/water over ambient storage of one week. At different mass fractions of up to 30 ppm, they found no precipitation after the one-week test, and to confirm this, they recorded the time-dependent absorbance spectra of the single- and multicomponent NFs. At low concentrations, they found overlapping in the spectra between the as-prepared samples and the same samples after seven days, while samples with higher concentrations exhibited some changes in absorbance.

Chen et al. [68] found that CQDs/PEG-200 prepared by microwave heating are stable over 30 days of ambient storage. They observed light sedimentation from optical images and very small changes in transmittance spectra. GO/water–EG NFs with GO of 0.06 wt% were irradiated with an ultraviolet lamp for 240–615 s to obtain a series of GO//water–EG NFs [88]. For comparison purposes, a reduced GO/water NF was also prepared at 0.02 wt% using PVP surfactant followed by UV irradiation for 340 s. After two months of ambient storage, GO/water–EG NFs obtained under irradiation durations of ≤465 s exhibited no sedimentation, while some sediments were observed at longer duration. When the temperature changed from 30 °C to 70 °C, GO/water–EG NFs at 0.06 wt% showed an increase in zeta potential, while the water-based GO NFs at 0.02 wt% showed the opposite, highlighting the effect of the base fluid used on the stability of NFs.

**Figure 10:**
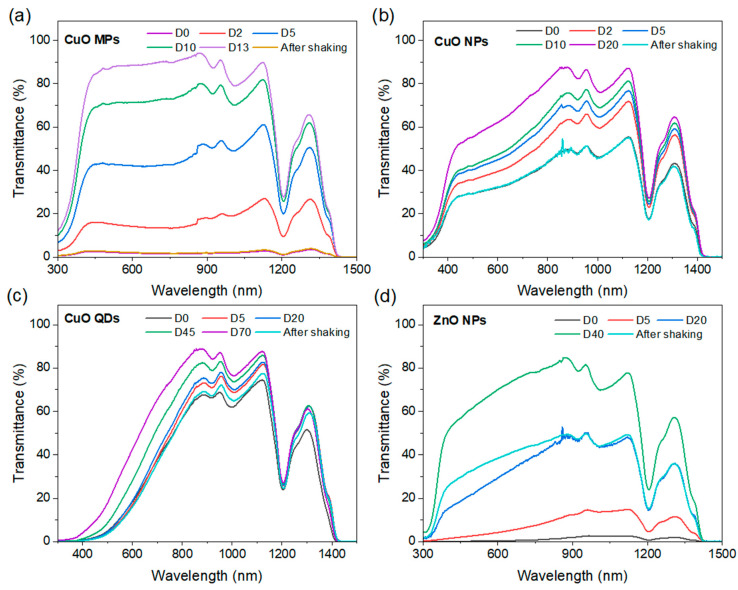
Transmittance variation in (**a**) CuO MPs, (**b**) CuO NPs, and (**c**) CuO QDs in addition to (**d**) ZnO NPs in EG across a wavelength range of 300–1500 nm. The transmittance measurements were performed in a static mode, i.e., without shaking, and were then manually shaken before the last measurement “After shaking”.

In a study on NF samples of two metal oxides of spherical Al_2_O_3_ NPs of 40 nm diameter and spherical CuO NPs of 100 nm diameter, as well as the combination of both (Al_2_O_3_-CuO NPs) in EG [85], the stability of the samples was tested over two weeks of ambient storage. NFs based on metal oxides offer interesting features for DASC applications due to their chemical inertness and thermal stability. Whilst many stoichiometric oxides exhibit very large bandgap values and low or no absorption in the solar spectral range, it is possible to produce metal oxide NPs with strong absorption also in the visible [29]. Pure CuO, for instance, deviating from the characteristics of most oxides, has a relatively low bandgap (~1.2 eV [113]), which is suitable for absorbing solar energy. However, the bandgap is impacted by quantum confinement and given the relatively large CuO Bohr radius (~28.27 nm [114,115]), the size of CuO NPs should be sufficiently large not to experience significant bandgap widening. Adversely, larger NPs will tend to affect the NF stability. This can be shown again by comparing the stability of the same CuO QDs, NPs, and MPs that we reported in Figure 8. In order to assess the long-term static stability of NFs, we have monitored the transmittance over days (Figure 10a–c), with the NF stored in a dark space at room temperature without any form of mixing or shaking (‘static stability’). The results show that while QDs (Figure 10c) exhibit superior stability compared to MPs (Figure 10a) or NPs (Figure 10b), their transmittance is relatively high, a consequence of quantum confinement and widening of the bandgap (to 2.2 eV [116,117]). As the cumulative transmittance increases with the size of the CuO particles, the static stability worsens and the best compromise between optical performance and stability should be found. However, static stability tests can be viewed as too severe for DASC applications, considering that NFs are generally in some form of motion when used in DASCs. Here, we show that after a simple manual shaking (“after shaking” in Figure 10a–c), the transmittance of all different types of CuO particles return to the values measured at the start (D0).

The manipulation of the morphology or chemical composition of metal oxide NPs also offers other opportunities to enhance the combined optical-stability performance of NFs. In Figure 10d, we report the stability of NFs produced with oxygen-deficient ZnO NPs. The results show substantial improvements in the optical properties with very low cumulative transmittance at day 0 (D0) compared to the expected high transparency of ZnO NPs, due to the very high ZnO bandgap. While the stability of these NFs with ZnO NPs requires some optimization, these still outperform other CuO-based NFs after 20 days.

### Performance of Nanofluid-Based DASCs

**Figure 11:**
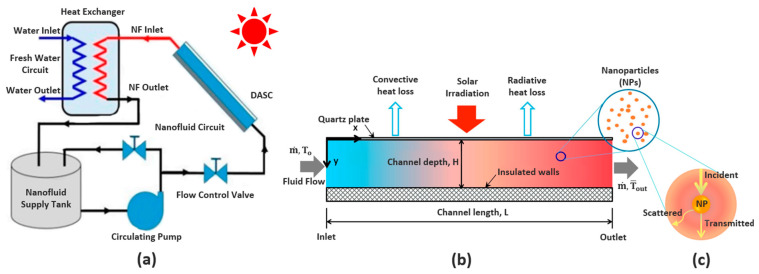
DASC schematics; (**a**) simplified closed-loop system for heat transfer. Adapted from [24], (**b**) irradiation and heat loss sources. Adapted from [118], (**c**) nanoparticle and solar irradiation interaction.

DASCs are used in solar thermal systems, as illustrated in Figure 11a. The overall performance of DASC systems is impacted by various losses. Before reaching the nanofluid surface, the incident solar radiation intensity ($I_{o}$) is reduced by reflection at the surface of the transparent glass layer (${I_{ref}}_{glass}$) as well as absorption through it (${I_{abs}}_{glass}$). It is worth mentioning that despite this loss in incident radiation intensity, the glass layer plays an important role in improving the overall performance of the DASCs by reducing the thermal energy loss to the surrounding, which is relatively high compared to the loss in radiation intensity. The amount of solar radiation reaching the nanofluid ($I_{nf}$) undergoes an STC process through which the solar radiation is absorbed and scattered while travelling through the NF, as shown in Figure 11c (see also Figure 3). Here, we refer to the total radiation absorbed as ${I_{abs}}_{nf}$, while we use ${I_{sc}}_{nf}$ to refer to the amount of light that is scattered or that escaped the NF without re-absorption. Furthermore, thermal energy is then generated ($Q_{gen}$) from the absorbed radiation via an STC process. With the increase in the NF temperature above the surrounding temperature, a portion of the generated thermal energy is lost to the surrounding, as shown in Figure 11b, by convection (${Q_{loss}}_{conv}$) and radiation (${Q_{loss}}_{rad}$). Thus, the thermal energy gain within the NF, i.e., the net thermal energy (${Q_{net}}_{nf}$), can be obtained using the energy balance as follows:

(8) $${Q_{net}}_{nf}=I_{o}-\left[\left({I_{ref}}_{glass}+{I_{abs}}_{glass}\right)+{I_{sc}}_{nf}+\left({Q_{loss}}_{conv}+{Q_{loss}}_{rad}\right)\right]$$

The net thermal energy can be also determined at a given mass flow rate ($\dot{m}$), specific heat capacity ($c_{p}$), and temperature rise (ΔT) of the NF:

(9) $${Q_{net}}_{nf}=\dot{m}\times c_{p}\times \Delta T$$

The overall efficiency of a DASC system, ($\eta_{DASC}$), can be determined from the ratio of the net thermal energy stored by the nanofluid and the incident solar radiation, as follows:

(10) $$\eta_{DASC}=\frac{{Q_{net}}_{nf}}{I_{o}}$$

Other aspects to be considered when evaluating the overall efficiency of a DASC system include the diffraction and reflection of light at the outer and inner surfaces of the glass layer, as well as at the interfaces between the NF and the DASC walls that contain the NF, and the variation in the ambient temperature.

**Figure 12:**
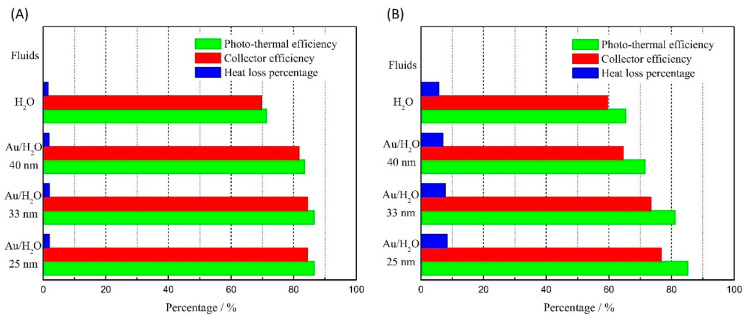
Photo-thermal and collector efficiencies as well as the heat loss of different NFs in (**A**) cube-shaped and (**B**) flat DASCs (adapted from [58]).

DASCs can take a variety of geometries/shapes to achieve the desired performance within a defined range of operating temperatures. For instance, Chen et al. [58] described the effect of DASC design on the plasmonic absorption-induced STC of Au NPs/water NFs in a static mode under simulated solar radiation of 450 W/m^2^. They tested flat and cubic DASCs made of polymethyl methacrylate (PMMA). The flat- and cubic-shaped DASCs were designed with inner dimensions of 100 mm × 100 mm × 6 mm and 50 mm × 50 mm × 48 mm, respectively, and both were thermally insulated. As depicted in Figure 12, they found that at a concentration of Au NPs of as low as 0.000008 wt%, the flat-shaped DASC system improved the STC efficiency by 21.3%, which was higher than that of the cube-shaped DASC (19.9%) at the same concentration. They also included the impact of variation in the size of Au NPs (25 nm, 33 nm, and 40 nm) to see if this can alter the performance results of both shapes. The STC efficiency in the flat DASC increased by reducing the NP size, while this size reduction did not influence the cube-shaped DASC. However, the cube-shaped DASC exhibited higher overall efficiency since the flat-shaped DASC exhibited higher heat loss. From this, we can see that the performance of DASC systems can be tuned by their size and shape.

The effect of NP concentration and solar radiation intensity were studied by Wang et al. [77]. They investigated the effect of these parameters on the absorption and STC performance of EG-based TiN NFs. They used TiN NPs at various concentrations (0.001–0.01 wt.%) in static mode in a thermally insulated (with aerogel) DASC made of quartz with a radius and height of 1.75 cm and 4 cm, respectively. They irradiated the samples with solar radiation of intensity up to two suns (2000 W/m^2^) and studied the effect of two different directions of the radiation on the DASC performance: side and bottom. The absorption performance of the NFs increased by ~50% with the increase in temperature from 0 °C to 60 °C. At a concentration of 0.003 wt.%, the bottom irradiation mode achieved a conversion efficiency of 45% higher than that of side irradiation. However, the latter saved nearly 40% of the time required to reach steady-state temperature when compared with the side radiation mode. In addition, both radiation direction modes exhibited uniform temperatures over the NF depth of 1 cm. Ag/water, Fe_3_O_4_/water and graphite/water were tested in a dynamic mode (fluid circulation) [80] in a rectangular channel made of stainless steel (12 mm × 5 mm × 2 mm), with the top glazing made of low-reflectance borosilicate glass of 4 mm and the inner surface of the bottom wall covered with reflective steel. The DASC was thermally insulated by a 3 cm thick Styrofoam with a thermal conductivity of 0.03 W/m K. At varying simulated solar radiation of 600 W/m^2^, 800 W/m^2^ and 1000 W/m^2^, Fe_3_O_4_/water showed the highest outlet temperature and collector efficiency at all radiation flux values followed by graphite/water, with Ag/water being the lowest.

Fe_2_O_3_/water NFs at two concentrations of 0.5 wt% and 2 wt% were investigated by Balakin et al. [74] in a dynamic mode with Reynolds numbers up to 1000 in a DASC of 400 mm long transparent glass tube with an internal diameter of 4 mm. The NFs reached a maximum thermal efficiency of 65% at the highest concentration of 2 wt%, while the lowest concentration of 0.5 wt% achieved 8%. In another similar work [75] Fe_2_O_3_/water NFs were used in an evacuated tubular DASC made of a borosilicate glass tube of 140 cm in length and containing a smaller glass tube of 2.5 cm and 4 cm inner and outer diameters, respectively. The flow rate was 400 mL/min (Reynold number of 340) and a surface area of 0.055 cm^2^ was exposed to solar radiation. At a 4 cm sample depth, Fe_2_O_3_/water at 0.02 vol% absorbed all incident radiation while the other NF absorbed 17% less at the same volume fraction. In terms of STC efficiency, Fe_2_O_3_/water confirmed its better performance over Fe_3_O_4_/water by a maximum efficiency of 73%, which is higher by 22% than the maximum efficiency reached by Fe_2_O_3_ NF at 0.02 vol%. CQDs of less than 10 nm in diameter prepared by microwave heating [68] were dispersed in PEG-200 and investigated in a static mode under simulated solar radiation of 1–4 sun for 60 min under continuous magnetic stirring in a DASC made from a quartz vessel of 3 cm × 3 cm × 3 cm with a sponge plate on the top to reduce sample evaporation. The results revealed that extending the microwave heating duration has an advantageous impact on STC efficiency. In addition, the efficiency reached its maximum of 81% when exposed to 1 sun and microwave heating was 24 min, which is triple the efficiency of the base fluid PEG-200 (27%).

Guo et al. [50] produced NFs with GO (0.5–5 μm diameter), CNTs (−5 nm and 8–15 nm inner and outer diameters), and Ti_3_C_2_ at concentrations from 0.001 wt% to 0.04 wt% for investigating their STC performance in a static mode in a quartz DASC of 2.5–4 cm height. The collector was covered with a sponge, except for the top part, and it was exposed to varying solar concentrations from 1 sun to 2.5 suns. Ti_3_C_2_ outperformed both GO and CNTs by achieving an STC efficiency of 20% and 29% higher. The STC efficiency of the 0.04 wt% Ti_3_C_2_ NF was 2.7 times that of the pure working fluid when irradiated for 6500 s at a solar concentration ratio of 2.5 with a DASC height of 2.5 cm. In terms of concentration effect, they found that 0.02 wt% was the optimal particle concentration since no significant enhancement in performance was found at higher concentrations. Zheng et al. [79] studied the solar absorption and STC efficiency of a single-component NF of CNT/water (5–10 nm and 20–30 nm inner and outer diameters and 10–30 μm length) and a muti-component NF of CNT–TiN/water (TiN NPs are 20 nm in diameter) at mass fractions of 10 ppm, 20 ppm, and 30 ppm. The DASC that they used for this work was a cylinder made of acrylic with a 15 mm height, 26.4 mm inner diameter and 2 mm wall thickness. To reduce heat losses, they covered the DASC top with a 1 mm quartz glass of over 90% light transmittance and they also used an aluminium mirror at the bottom to reflect transmitted light back to the NFs. In a static mode and under one sun radiation intensity, the STC efficiency of the single- and multicomponent NF reached 25% and 26%, respectively, which is higher by 8.7% and 13.0%, respectively, compared to that of the base fluid. The increase in STC efficiency by the CNT–TiN/water over the single-component NF was attributed to the plasmonic absorption of TiN NPs. The increase in mass fraction above 10 ppm increased scattering by TiN NPs, which negatively impacted the efficiency.

**Figure 13:**
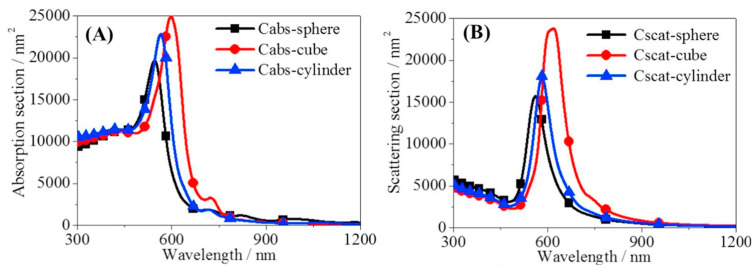
Optical properties of different Au nanostructures; (**A**) absorption section, and (**B**) scattering section. Adapted from [120].

The physical shape of NPs plays a crucial role in determining the performance of NFs. Nanoparticles with different shapes have distinct physicochemical properties, such as surface area, surface energy, and surface charge. These properties affect the absorption, stability, and thermal conductivity of NFs. Maheshwary et al. [119] explored the impact of NP shapes on the thermal conductivity and stability enhancement of NFs. The study investigated the thermal conductivity and stability of five different water-based NFs, CuO, MgO, TiO_2_, ZrO_2_, and Al_2_O_3_, with different shapes of spherical, cubic, and rod-shaped NPs. All NFs were prepared with the same concentration of 2.5 wt%. It was revealed that the NF of cubic Al_2_O_3_ showed the highest thermal conductivity over the base fluid by 3.13 times. However, the cubic-shaped NPs exhibited lower stability compared to spherical and rod shapes. Chen et al. [120] reported that cubic Au NPs produce higher solar heating and higher absorption than spherical and cylindrical NPs, as shown in Figure 13. Additionally, Wang et al. [121] indicated that the absorption, in terms of both magnitude and bandwidth, of the cubic core–shell carbon Au NPs was better than spherical NPs.

Using mixed NP morphologies can enhance the performance of NFs in terms of absorption and thermal conductivity. Mixed Au NPs with different shapes were used by Duan et al. [122] to prepare a plasmonic water-based NF and study this for DASC applications. A plasmonic-blended NF was created by mixing Au NPs of three distinct shapes (spherical, rod, and star-shaped) in water. Both experimental and numerical studies showed that the optical and thermal properties of blended NFs indicate that the extinction spectra of Au blended NFs were wider in comparison to single-component nanofluids. Moreover, the photothermal properties of these three mixed shapes of NPs showed a higher temperature rise and higher heat collection ability, and the experimental results aligned with the numerical analysis findings.

NFs have shown promising potential in the performance enhancement of DASCs by increasing the heat transfer rate and increasing the absorption of the solar spectrum. However, there are some limitations and disadvantages that need to be considered before implementing NFs in DASCs. One of the primary concerns is the stability of NFs since NPs tend to agglomerate and settle down over time and negatively affect the homogeneity of the fluid properties. Consequently, the usability of NFs will be limited for some applications, especially those that demand a higher concentration of NPs [63,123]. Corrosion is also another challenge, as some NPs can corrode the components of the solar collector, leading to material degradation and reduced efficiency [92]. The viscosity of NFs is also a problem, as NFs have higher viscosities compared to traditional heat transfer fluids, which means that they encounter greater resistance when flowing through a system. As a result, the pressure drop in the system increases and more power is needed to maintain the flow rate [124]. Overall, while the deployment of NFs for DASCs still presents some challenges, scientific and technological progress has shown that these can be overcome in the future.

## 6. Conclusions: Challenges, Research Gaps and Future Perspectives

From the literature reviewed above, researchers have clearly made commendable progress in the development of efficient and stable NFs for DASCs. However, research work is still required to bring this new technology from lab experiments to large-scale production and attract investments from the industrial sector. The commercialization of NF-based DASCs will depend on completely resolving issues such as the instability of NFs, high costs and the synthesis complexity of nanomaterials [125], increased pumping power and the appearance of mechanical erosion and chemical corrosion due to deposition of NPs on the inner surfaces of flow channels [126], in addition to possible environmental concerns [127,128]. An in-depth understanding is required for sedimentation, agglomeration, morphological transformations, and the chemical deterioration of NPs in base fluids over long periods of operation, at elevated temperatures, and under thermal and solar cycling processes, as these factors are all critical to the optical, thermal, and rheological properties of NFs. Many optimization avenues are available and, for instance, NP loading, flow conditions, DASC system geometry, and NP chemical/structural properties can all be studied and optimized to overcome the current challenges.

High concentrations of NPs increase scattering at the surface, increasing radiative losses, which further increase with particles of large sizes, as well as higher heat losses due to the overheating of the NF surface [128]. At very low NP loadings, e.g., less than 0.1 vol%, such limitations may not affect the applicability of NFs in DASCs. Another benefit of using very low particle loadings in DASCs is that it alleviates the problem of nanomaterials synthesis, which can be costly and sophisticated in some cases [129]. However, because low particle loading may compromise the ability of NFs to maintain high solar absorption and thermal conduction of the NFs, further research work is still needed to develop innovative solutions for simple and cost-effective techniques for both the synthesis of nanomaterials and the preparation of stable NFs. Since DASCs are a green technology that depends on solar energy, it is highly important to use NFs with no environmental impacts. In some cases, NFs may have environmental and toxicological effects [130,131,132], hence the selection of appropriate size, surface properties, and chemical composition is also important.

Solar thermal energy conversion technologies can greatly benefit from ongoing and future NF research. NF fundamental and physical properties can be reliably assessed in a laboratory setting and can provide valuable information in the development of full DASC systems aided by computer simulations. The availability of sophisticated synthesis methodologies in combination with fundamental materials science investigations can produce very high specification NFs at sufficiently low costs for the full deployment of NF-based DASCs.

## Data Availability

The data presented in this study are available on request from the corresponding author.
